# Biofabricated Silver Nanoparticles Act as a Strong Fungicide against *Bipolaris sorokiniana* Causing Spot Blotch Disease in Wheat

**DOI:** 10.1371/journal.pone.0097881

**Published:** 2014-05-19

**Authors:** Sandhya Mishra, Braj Raj Singh, Akanksha Singh, Chetan Keswani, Alim H. Naqvi, H. B. Singh

**Affiliations:** 1 Department of Mycology and Plant Pathology, Institute of Agricultural Sciences, Banaras Hindu University, Varanasi, India; 2 Centre of Excellence in Materials Science (Nanomaterials), Department of Applied Physics, Z. H. College of Engineering and Technology, Aligarh Muslim University, Aligarh, India; University of Wisconsin - Madison, United States of America

## Abstract

The present study is focused on the extracellular synthesis of silver nanoparticles (AgNPs) using culture supernatant of an agriculturally important bacterium, *Serratia* sp. BHU-S4 and demonstrates its effective application for the management of spot blotch disease in wheat. The biosynthesis of AgNPs by *Serratia* sp. BHU-S4 (denoted as bsAgNPs) was monitored by UV–visible spectrum that showed the surface plasmon resonance (SPR) peak at 410 nm, an important characteristic of AgNPs. Furthermore, the structural, morphological, elemental, functional and thermal characterization of bsAgNPs was carried out using the X-ray diffraction (XRD), electron and atomic microscopies, energy dispersive X-ray (EDAX) spectrometer, FTIR spectroscopy and thermogravimetric analyzer (TGA), respectively. The bsAgNPs were spherical in shape with size range of ∼10 to 20 nm. The XRD and EDAX analysis confirmed successful biosynthesis and crystalline nature of AgNPs. The bsAgNPs exhibited strong antifungal activity against *Bipolaris sorokiniana*, the spot blotch pathogen of wheat. Interestingly, 2, 4 and 10 µg/ml concentrations of bsAgNPs accounted for complete inhibition of conidial germination, whereas in the absence of bsAgNPs, conidial germination was 100%. A detached leaf bioassay revealed prominent conidial germination on wheat leaves infected with *B. sorokiniana* conidial suspension alone, while the germination of conidia was totally inhibited when the leaves were treated with bsAgNPs. The results were further authenticated under green house conditions, where application of bsAgNPs significantly reduced *B. sorokiniana* infection in wheat plants. Histochemical staining revealed a significant role of bsAgNPs treatment in inducing lignin deposition in vascular bundles. In summary, our findings represent the efficient application of bsAgNPs in plant disease management, indicating the exciting possibilities of nanofungicide employing agriculturally important bacteria.

## Introduction

Agricultural production is constantly suffering from plethora of threats instigated by various classes of plant pathogens leading to huge economic losses. Consequently, innovative technologies are being introduced in modern agriculture to minimise such losses. Among such technological innovations, nanotechnology is gathering noteworthy considerations due to its robust applications in agriculture [1], [2]. In general, nanoparticles have attracted considerable attention in the fields of medicine, pharmaceuticals, cosmetics and electronics due to unique physical, chemical and biological properties [3]. Contemporarily, the development and application of biosynthesized nanoparticles has opened new avenues in agricultural research oriented to developing ecofriendly and effective means of controlling plant diseases. Most importantly, biological synthesis of silver nanoparticles (AgNPs) has offered a consistent, non toxic and ecofriendly approach for plant disease management due to their strong antimicrobial properties [4]–[6].

Antimicrobial property of AgNPs has been exploited to a great extent against a broad range of human pathogens [7]–[10]. However, the full potential of AgNPs is still largely unexplored for crop protection and presently there has been a growing interest to utilize their antimicrobial property for plant disease management. Earlier findings have advocated the toxicity of AgNPs on fungal hyphae and conidial development [11], [12]. In recent years, *in vitro* assays conducted by Krishnaraj and colleagues [13] and Gopinath and Velusamy [14] showed the strong inhibitory effects of biosynthesized AgNPs against various fungal plant pathogens. In another study, AgNPs synthesized using cow milk, have also shown strong antifungal activity against a range of phytopathogens [15].

The above reports clearly provide substantial basis for exploring the possibility of an efficient application of AgNPs in controlling phytopathogens under *in vitro* conditions. However, a rigorous *in vivo* assessment is essential to authenticate their functional applications under field conditions. Furthermore, considering the significance of agriculturally important microbes, their use for synthesizing AgNPs with strong antimicrobial properties can certainly provide an alternate means for plant protection. Therefore, the prime objective of the present study is to use a plant growth promoting rhizobacterium (PGPR) for synthesis of AgNPs and to evaluate the biocontrol potential of bsAgNPs both under *in vitro* and *in vivo* conditions. In this regard, we report a newly isolated *Serratia* sp. BHU-S4 from agricultural soil that has the potential to synthesize AgNPs with strong antifungal activity against *Bipolaris sorokiniana*, the spot blotch pathogen of wheat. Spot blotch is considered as one of the most dreadful disease causing 20% yield loss in South Asia [16], while in India, yield loss has been estimated upto 22% [17]. The increasing threat of spot blotch pathogen leads to 80% disease severity and the losses could be as high as 100% under severe conditions [18], [19]. Hence, the present study was initiated with an aim to determine the efficiency of biosynthesized AgNPs (denoted as bsAgNPs) in controlling infection of *B. sorokiniana* which causes spot blotch disease in wheat. Apart from *in vitro* assays, plant test under greenhouse condition was also conducted to ascertain the efficiency of bsAgNPs in controlling disease incidence.

## Materials and Methods

### Isolation and Identification of Bacterial Strain BHU-S4

Bacteria were isolated from soil samples collected from agricultural fields of the Institute of Agricultural Sciences, Banaras Hindu University campus, Varanasi (25°20′N latitude & 83°01′E longitude), Ramnagar (25°18′N latitude & 83°02′E longitude), Mirzapur (25°10′N latitude & 82°37′E longitude) and Chunar (25°07′N latitude & 82°54′E longitude), India. The soil samples were subjected to serial dilution and plated on Nutrient Agar (NA) medium. After incubation at 30°C for 24 h, bacterial colonies were subcultured and further purified on NA. On the basis of rapid reduction of AgNO_3_ into AgNPs, the bacterial strain BHU-S4 was selected for further studies. The preliminary characterization of the bacterial strain BHU-S4 based on physiological and biochemical characteristics was carried out according to Bergey’s Manual of Determinative Bacteriology [20]. Further authentic taxonomic characterization of bacterial strain BHU-S4 was done by 16S rDNA gene sequence homology and phylogenetic analysis [21], [22].

The qualitative plant growth promoting activities of the strain BHU-S4 were also ascertained. The inorganic phosphate solubilizing ability was determined using NBRIP-BPB medium as described earlier [23]. The indole acetic acid (IAA) production was determined according to the method described by Brick et al. [24] and siderophore production was tested using Chromazural S (CAS) assay as described by Dwivedi et al. [25].

### Extracellular Biosynthesis of AgNPs by *Serratia* sp. BHU-S4

The bacterial strain BHU-S4 was inoculated in Nutrient Broth (NB) medium and incubated at 30°C for 48 h to attain the early stationary phase. The culture supernatant was obtained by centrifugation at 10,000 rpm for 10 min and transferred to another sterile conical flask. The fresh stock of silver nitrate (AgNO_3_) was prepared in sterile distilled water and added to the culture supernatant at the final concentration of 1 mM. The conical flask was incubated in dark and the synthesis of AgNPs was monitored by visual color change from yellow (original color of culture supernatant) to dark brown (color of culture supernatant after adding AgNO_3_) [14], [26]. The bsAgNPs were air dried in sterile conditions and obtained in the form of powder for further studies.

### Characterization of bsAgNPs

The optical, structural, morphological, elemental, functional and thermal characterization of the bsAgNPs was carried out using the UV–visible spectrophotometery, X-ray diffraction (XRD), electron and atomic microscopies, energy dispersive X-ray (EDAX) spectrometer, Fourier transform infrared (FTIR) spectroscopy and thermogravimetric analyzer, respectively. In order to ascertain the optical characteristics of bsAgNPs in reaction solution, the absorption spectrum was recorded by UV–visible spectrophotometer (Perkin Elmer Life and Analytical Sciences, CT, USA) in the wavelength range of A_200_ to A_600 nm_ using quartz cuvette [27]. The structural characteristic of bsAgNPs powder sample was recorded on MiniFlex II benchtop XRD system (Rigaku Corporation, Tokyo, Japan) operating at 40 kV [22]. The scanning electron microscopy (SEM) was carried out using fine powder of the bsAgNPs on a carbon tape in JSM 6510LV scanning electron microscope (JEOL, Tokyo, Japan) at an accelerating voltage of 20 kV. The elemental analysis of bsAgNPs was done using Oxford Instruments INCAx-sight EDAX spectrometer equipped SEM [28]. The transmission electron microscopy (TEM) of bsAgNPs was carried out on JEOL 100/120 kV TEM (JEOL, Tokyo, Japan) with an accelerating voltage of 200 kV [29]. For atomic force microscopy, a thin film of bsAgNPs was prepared on the borosilicate glass slide to analyse the surface morphology. The prepared thin film was analysed on the atomic force microscope (AFM; Innova SPM, Veeco) in the tapping mode. The commercial etched silicon tips were used as scanning probes with typical resonance frequency of 300 Hz (RTESP, Veeco). The microscope was placed on a pneumatic anti-vibration desk, under a damping cover and the analysis was done using SPM Lab software [30], [31]. The images of electron microscopies of EDAX and AFM were obtained and converted into an enhanced meta file format. For the functional characterization of the bsAgNPs, the powder was mixed with spectroscopic grade potassium bromide (KBr) in the ratio of 1∶100 and spectra was recorded in the range of 400–4000 wavenumber (cm^−1^) on Perkin Elmer FT-IR spectrometer Spectrum Two (Perkin Elmer Life and Analytical Sciences, CT, USA) in the diffuse reflectance mode at a resolution of 4 cm^−1^ in KBr pellets [32]. The functionalization and thermal stability of the bsAgNPs was investigated by thermogravimetric analysis (TGA) (Perkin Elmer Pyris 1 TGA Thermogravimetric Analyzer) at a heating rate of 10°C/min under nitrogen atmosphere [33].

### Antagonistic Potential of bsAgNPs against *Bipolaris sorokiniana*

#### Cavity slide experiment

A cavity slide experiment was designed in triplicate to evaluate the antagonistic potential of bsAgNPs against *B*. *sorokiniana*. Conidial suspension of *B*. *sorokiniana* was prepared in sterile distilled water from 10 days old pure culture under aseptic conditions. The conidial suspension (40 µl) was mixed with different concentrations (2, 4 and 10 µg/ml) of bsAgNPs and filled in each cavity. The control set was maintained separately by filling the cavity with conidial suspension without bsAgNPs. Three replicates each for control and treated set were maintained. The cavity slides were incubated at 25±2°C in moist chambers (Petri dishes, 90 mm diameter, containing blotting papers soaked in sterile distilled water) for 24 h. After incubation period, slides were examined under microscope to evaluate inhibitory effect of bsAgNPs on conidia germination of *B*. *sorokiniana*.

#### Detached leaf assay

Wheat leaves were washed with sterile distilled water, surface sterilized by dipping into 1% sodium hypochlorite for 30 sec and further rinsed 5 times with sterile water under aseptic conditions. Leaves were placed in a petriplate containing 0.5% agar and inoculated (5 spots per leaf) by pipetting 10 µl droplets of *B*. *sorokiniana* conidial suspension mixed with 4 µg/ml of bsAgNPs. Leaves spotted with 10 µl droplets of *B*. *sorokiniana* conidial suspension served as positive control.

#### Greenhouse experiment

Plant test was performed under greenhouse conditions using wheat as a host plant. Wheat (*Triticum aestivum*) var. HUW-234 seeds were sown in pots (9 cm diameter) containing sterilized soil and irrigated with water to maintain 20% soil moisture. The plant test consisted of following treatments: control (C), pathogen challenged control (BC), pathogen challenged + bsAgNPs treated (B4). For each treatment, six pots were used and ten seeds were sown in each pot. After 15 days of growth, treatment of pathogen and bsAgNPs was given. *B*. *sorokiniana* conidial suspension (containing 10^9^ conidia) was prepared in sterile distilled water. Conidial suspension was mixed with bsAgNPs suspension in 1∶1 ratio and sprayed over the plants while plants sprayed with conidial suspension alone served as pathogen challenged control. Plants without conidial suspension and bsAgNPs treatments served as healthy control. Pathogen challenged plants were covered with polybags for 48 h to maintain the humidity level favoring pathogen infection. Data on plant growth parameters was recorded after 30 days of sowing and subjected to one-way ANOVA followed by Waller-Duncan posthoc test at *p*<0.05 using SPSS 16.0.

### SDS-PAGE Analysis

The total protein from wheat leaf samples was isolated using the protocol described by Sarkar et al. [34]. The concentration of protein was measured by the Bradford method [35]. An amount of 100 µg protein was resolved in 12% polyacrylamide gel and further stained with CBB G-250 (BioRad).

### Histochemical Staining

After harvesting the wheat plants, transverse sections of the stem were cut and mounted on a glass slide in aqueous solution of phloroglucinol in 20% HCl and observed under light microscope (Nikon DS-fi1, Japan) for lignin staining indicated by red-violet color [36].

### Ethics Statement

The locations for the collection of soil samples are very common agricultural fields, so it did not need specific permission. It is also confirmed that these fields did not involve any endangered or protected species. We used sampling procedures that did not harm the plant diversity of the locations.

## Results and Discussion

**Figure 1:**
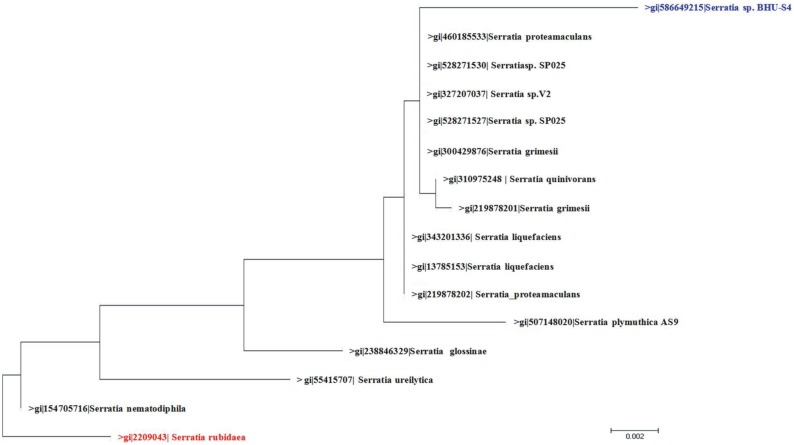
The phylogenetic tree of *Serratia* sp. BHU-S4 based on 16S rDNA sequence. The partial 16S rDNA sequence has been deposited in NCBI GenBank, nucleotide sequence database under the accession number KF863906. *Bar* 0.002 substitutions per site.

**Figure 2:**
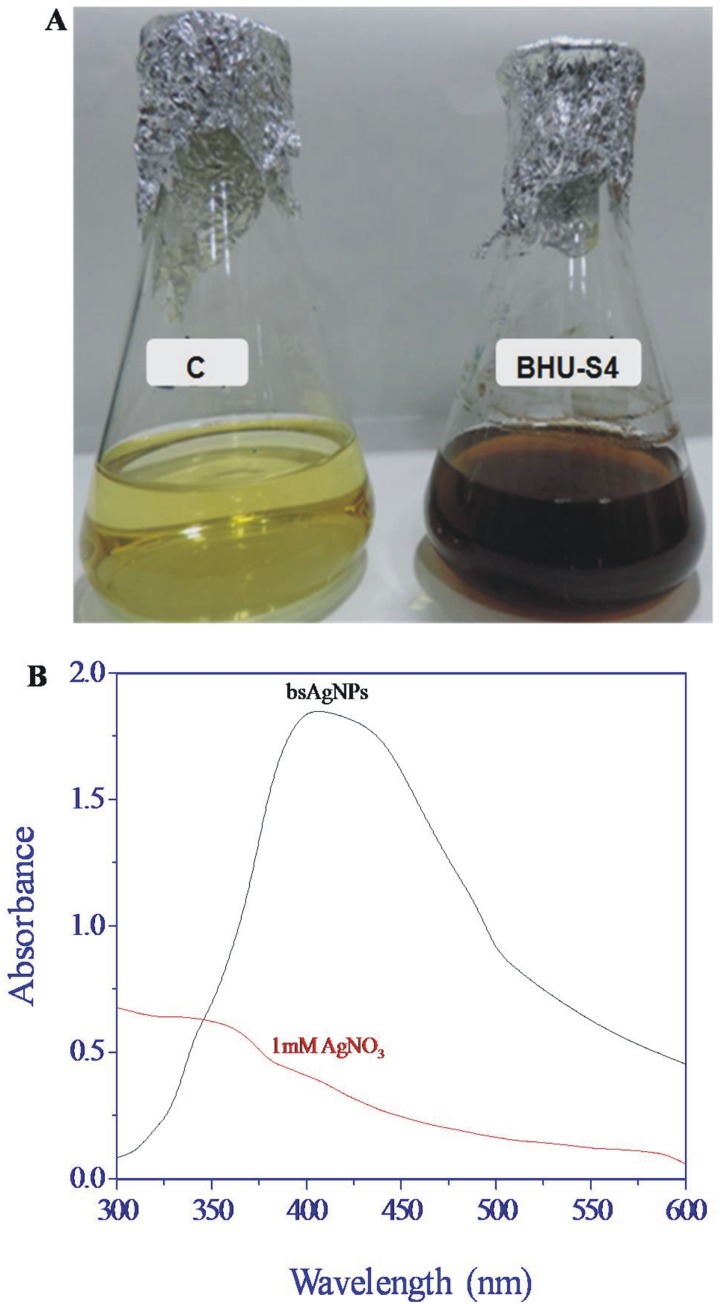
Biosynthesis of AgNPs by supernatant of *Serratia* sp. BHU-S4 (A). Culture supernatant without 1_3_ showed no color change (C) and after adding 1 mM AgNO_3_ showed visual color change from yellow to dark brown. (B) UV-visible absorption spectrum of bsAgNPs and 1 mM aqueous solution of AgNO_3_.

In the present study, the bacterial strain BHU-S4 was isolated from agricultural soil with the aim of exploiting its AgNPs synthesizing potential for agricultural purposes. Studies related to its plant growth promoting characters revealed its inorganic phosphate solubilization, IAA and siderophore production activities (Table 1). This bacterial strain was identified as a member of *Serratia* species by 16S rDNA gene sequencing, which has been deposited in NCBI GenBank (Accession Number: KF863906). The information obtained by the BLAST program and phylogenetic analysis indicated a close genetic relatedness of strain BHU-S4 with *Serratia* sp. and was therefore designated as *Serratia* sp. BHU-S4 (Figure 1). The species of genus *Serratia* are well known plant growth promoting rhizobacteria with biocontrol potential against various phytopathogens [37]–[39]. Subsequently, extracellular AgNPs synthesis by *Serratia* sp. BHU-S4 was performed and monitored by UV–visible spectrum that showed a strong and broad surface plasmon resonance (SPR) peak at 410 nm, which is a characteristic of AgNPs (Figure 2). However, this characteristic peak was absent in the aqueous solution of AgNO_3_. It is well known that due to Mie scattering, AgNPs exhibit absorption at the wavelength range of 390 to 420 nm [27], [40]. As evident from previous reports, the presence of single SPR peak indicates spherical shape of AgNPs which was further confirmed by XRD and electron microscopy [14], [41].

**Table 1:** Physiological and biochemical characteristics of *Serratia* sp. BHU-S4.

Characteristics	*Serratia* sp. BHU-S4
Gram reaction	G−ve
Auxin production	+
Phosphate solubilization	+
Siderophore production	+
Catalase	+
Proteolytic activity	+
Cellulolytic activity	+

### Characterization of Biosynthesized AgNPs

**Figure 3:**
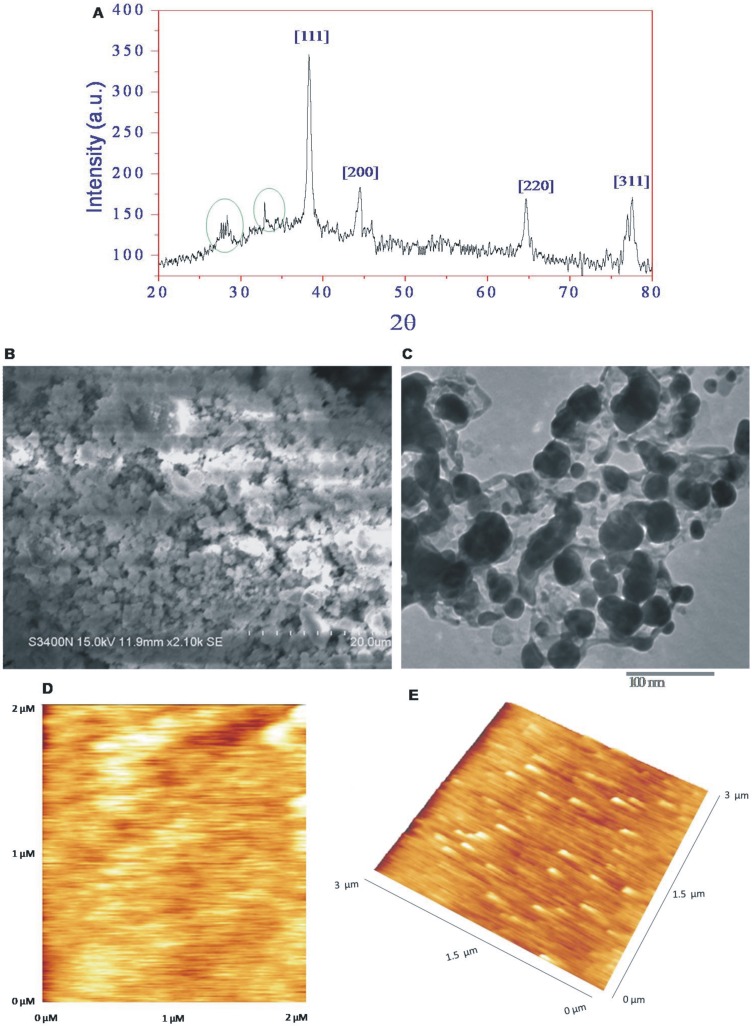
X-ray diffraction pattern (A), SEM (B) and TEM (C) micrographic images of bsAgNPs by *Serratia* sp. BHU-S4. AFM micrographs 2D (D) and 3D (E) illustrating the nanostructure of bsAgNPs.

**Figure 4:**
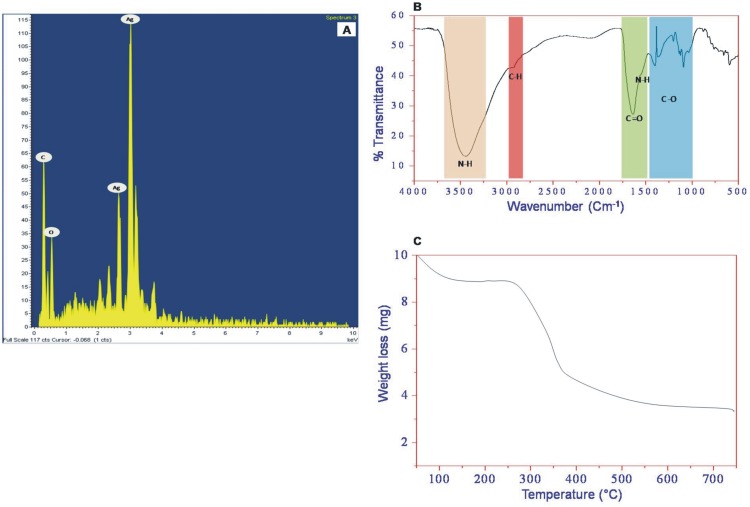
EDAX spectrum (A), FTIR spectrum (B) and TGA graph (C) of bsAgNPs by *Serratia* sp. BHU-S4.

The crystalline nature of bsAgNPs was characterized by XRD with Cu Kα radiation (λ = 0.15418 nm). The data revealed that the well resolved Bragg reflections were obtained at 2θ = 38.4°, 44.5°, 64.6° and 76.9° which correspond to the crystal lattice planes [111], [200], [220] and [311] of face centered cubic (*fcc*) structures of silver (JCPDS files No. 03-0921), respectively (Figure 3A). The unassigned Bragg reflections at 2θ = 28.1° and 32.9° marked with green circles are probably due to the crystallization of bioorganic phase that occurs on the surface of AgNPs. The XRD data indicated the successful synthesis of bsAgNPs in this study. The average crystallite size (*d*) of synthesized bsAgNPs was calculated following the Debye-Scherrer formula:
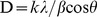
where, *k* = 0.9 is the shape factor, λ is the X-ray wavelength of Cu Kα radiation (1.54 Å), θ is the Bragg diffraction angle, and β is the full width at half maximum (FWHM) of the (111) plane diffraction peak. The average crystallite size was estimated to be 18.9 nm. The nanostructure of bsAgNPs was analyzed by SEM, TEM and AFM. The Figure 3B shows the scanning electron micrograph recorded from powder of bsAgNPs deposited on a carbon tape. The micrograph clearly demonstrated the aggregation of bsAgNPs into microparticles in powdered form. However, the TEM image showed a noticeable spherical morphology of bsAgNPs with a size range of ∼10–20 nm (Figure 3C), which was found to be consistent with the results obtained from XRD [42], [43]. TEM characterization revealed that bsAgNPs were smooth, well dispersed and crystalline in nature. However, agglomeration might be due to the presence of biological macromolecules on the surface of bsAgNPs. The AFM images also showed the irregular spherical surface morphology of bsAgNPs in the size range of ∼8 to 22 nm (Figure 3D and 3E), which is in accordance with the results obtained by XRD, SEM and TEM. The irregular spherical surface morphology of AgNPs observed in this study might be due to the biological macromolecules like proteins and enzymes present on the bsAgNPs surface. The elemental analysis using EDAX indicated the presence of mainly silver (Ag) in the bsAgNPs (Figure 4A), which reconfirmed that bsAgNPs were indeed metallic and crystalline in nature. Further, the occurrence of carbon and oxygen peaks revealed the presence of organic moieties on the surface of bsAgNPs.

The surface functionalization and stabilization of the bsAgNPs by the biological macromolecules present in the *Serratia* sp. BHU-S4 culture supernatant was confirmed by FTIR spectroscopy. The FTIR spectrum recorded from the dried powder of bsAgNPs is shown in Figure 4B. The result suggests the release of extracellular proteins in the *Serratia* sp. BHU-S4 strain culture supernatant which are primarily responsible for the effective synthesis and stabilization of the AgNPs. The amide linkage between consecutive amino acid residues in proteins provides a well-known signature in infrared region of the electromagnetic spectrum. The vibration bands position at 3436.52 cm^−1^ and 2942.73 cm^−1^ were assigned to the stretching vibrations of primary and secondary amines, respectively. The corresponding bending vibrations were observed at 1641.93 cm^−1^ assigned to chelated –C = O or –OH group from –COO group in the extracellular proteins. Besides, the two bands observed at 1387.92 cm^−1^ and 1087.45 cm^−1^ can be assigned to the C–N stretching vibrations of aromatic and aliphatic amines, respectively. The FTIR data suggested the presence of amine and carbonyl groups in proteins present in the *Serratia* sp. BHU-S4 strain culture supernatant which act as reducing and stabilizing agents through the capping of bsAgNPs [44], [45]. Thus, the higher stability of the bsAgNPs could be attributed to the complex nature of the *Serratia* sp. BHU-S4 strain culture supernatant [27], [46]–[48].

The functionalization and stabilization of bsAgNPs by the biological macromolecules present in the *Serratia* sp. BHU-S4 strain culture supernatant was further confirmed by TGA. This analysis was performed under inert N_2_ atmosphere and obtained thermogram is shown in Figure 4C. The thermogram revealed the two steps of weight loss in the temperature range of 50°C to 700°C. The first step weight loss (12%) was observed at ∼100 to 200°C due to evaporation of adsorbed water molecules present in the bsAgNPs sample. The second step weight loss (51%) observed in the temperature range of ∼300 to 450°C was a consequence of desorption of biological macromolecules like proteins and enzymes present on the surface of the bsAgNPs. It is evident from the above data that bsAgNPs were successfully synthesized using the culture supernatant of the *Serratia* sp. BHU-S4.

### Biocontrol Potential of Biosynthesized AgNPs against *B. sorokiniana*

**Figure 5:**
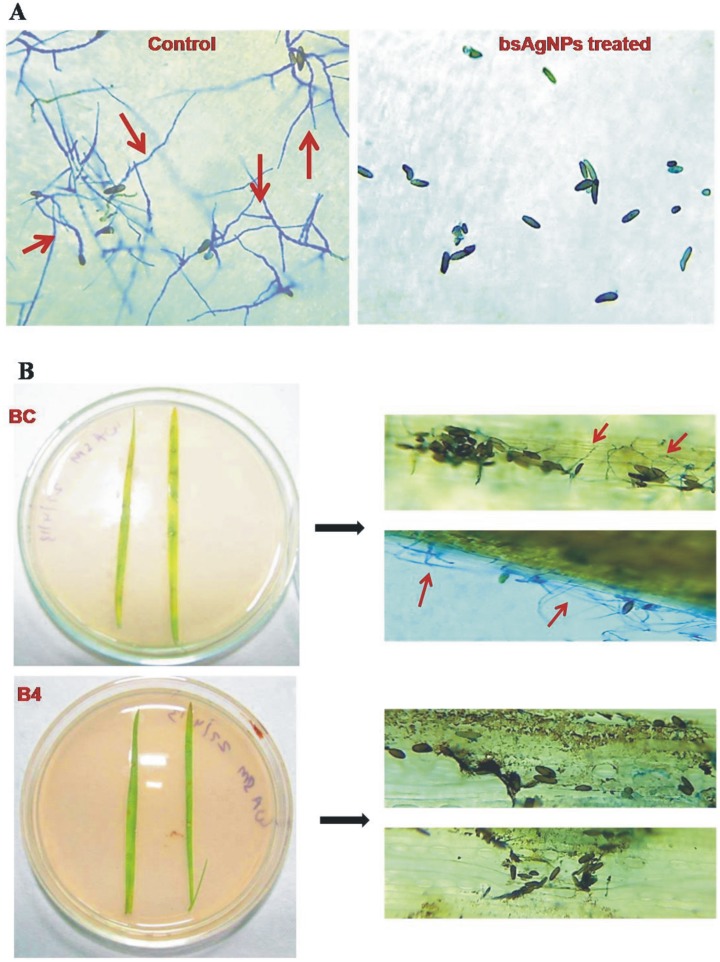
Inhibitory effects of bsAgNPs by *Serratia* sp. BHU-S4 on conidial germination of *B. sorokiniana* after 24 h as determined by cavity slide experiment. Conidial germination in control set (without bsAgNPs) was prominent as indicated by arrow (A). Detached leaf assay showing conidial germination over leaf surface in pathogen treated leaf (BC), while in presence of bsAgNPs (B4) conidial germination was totally inhibited (B).

The bsAgNPs by *Serratia* sp. BHU-S4 were also examined for their antifungal activity against *B. sorokiniana,* the spot blotch pathogen of wheat under *in vitro* and *in vivo* conditions. *B. sorokiniana* causes serious foliar spot blotch disease in wheat leading to major yield loss [49]. The presence of thick-walled, elliptical conidia is the characteristic feature of this pathogen. The process of infection starts with conidial germination on the leaf surface followed by appresorium formation after which hyphae colonizes inter and intracellular leaf tissues [50]. Hence, the inhibitory effect of bsAgNPs on germination of *B. sorokiniana* conidia was tested under *in vitro* condition. Our results revealed that 2, 4 and 10 µg/ml concentrations of bsAgNPs accounted for total inhibition of conidial germination whereas in the absence of bsAgNPs, 100% conidial germination was recorded (Figure 5A). Furthermore, the effectiveness of bsAgNPs in obstructing the process of infection by *B. sorokiniana* was ascertained by detached leaf assay. Wheat leaves inoculated with conidial suspension of *B. sorokiniana* alone showed the characteristic disease symptom i.e. formation of spot blotch whereas on exposure to bsAgNPs and *B. sorokiniana* conidial suspension in combination failed to form spot blotch on wheat leaves which appeared to be as healthy as control leaves. This result was further confirmed after observing microscopic images which showed prominent conidial germination on wheat leaves inoculated with *B. sorokiniana* conidial suspension alone, while it was totally inhibited on treatment with bsAgNPs (Figure 5B).

**Figure 6:**
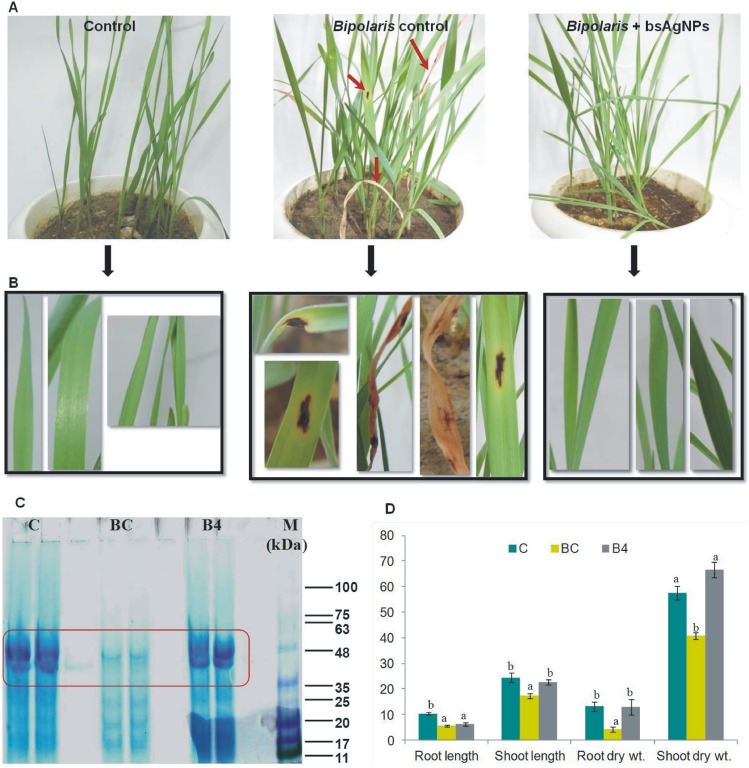
Efficacy of bsAgNPs in controlling pathogen attack under greenhouse conditions (A). Arrow indicates lesion developed as a result of *B. sorokiniana* infection. (B) Symptoms developed on leaves of *B. sorokiniana* infected plants (*Bipolaris* control). (C) SDS-PAGE profile of wheat leaf protein in different treatments. (D) Effect of different treatments on plant parameters are shown by bar graph. Control (C), *Bipolaris* control (BC); *Bipolaris* + bsAgNPs by *Serratia* sp. BHU-S4 (B4). Root length, shoot length (cm); root dry wt., shoot dry wt. (mg).

**Figure 7:**
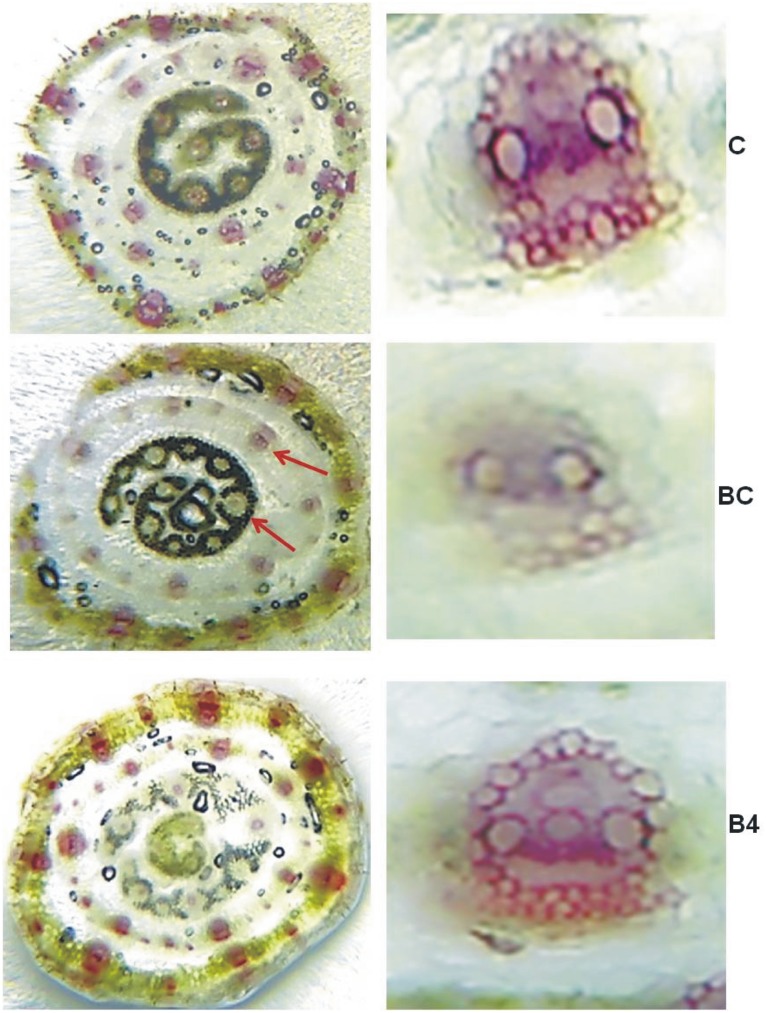
Effect of different treatments on lignification in wheat stem by histochemical staining. Control (C), *Bipolaris* control (BC); *Bipolaris* + bsAgNPs by *Serratia* sp. BHU-S4 (B4).

These findings validate the biocontrol potential of bsAgNPs against *B. sorokiniana* under *in vitro* conditions. However, for further validation, greenhouse experiment was also conducted where application of bsAgNPs strongly controlled *B. sorokiniana* infection in wheat (Figure 6A, B). Due to *B. sorokiniana* infection there was 46.73, 28.93, 68.47, 29.03% significant decrease in root length, shoot length, root dry wt. and shoot dry wt., respectively as compared to healthy control plants. It is interesting to note that decrease in plant growth after pathogen challenge was overcome when bsAgNPs treatment was given to the wheat plants that resulted into 11.55, 30.67, 60.83% increase in root length, shoot length and shoot dry wt. respectively, as compared to pathogen challenged plants (Figure 6D). Furthermore, to obtain an idea on the effect of different treatments on the wheat leaf proteome, we examined SDS-PAGE profile that cleraly showed major visible variations in protein banding pattern among different treatments (Figure 6C). The reduced intensity of protein bands justified the protein damage caused by *B. sorokiniana* infection. However, no protein damage occurred on bsAgNPs treatment as it showed banding pattern similar to control plants. Moreover, histochemical analysis of wheat stems of different treatments gave a clue about the defense strategy adopted by the plants against *B. sorokiniana* infection. Histochemical staining showed the pattern of lignification which is the main disease resistance factor as reported by Mandal and Mitra [51]. Notable difference in lignin deposition among different treatments was observed. Maximum lignification of vascular bundles was observed in control (C) and pathogen challenged treated with bsAgNPs plants (B4) while minimum lignin deposition was found in pathogen challenged plants (BC) (Figure 7). It is evident from previous reports that lignin deposition plays a crucial role in plant development as well as disease resistance by forming a physical barrier against pathogen attack [52], [53]. In addition, the content and composition of lignin is also reported to vary when plants are exposed to various stresses. Consequently, we hypothesize that the treatment of bsAgNPs enhanced lignification which could have worked as a hindrance against pathogen attack in plants treated with pathogen and bsAgNPs in combination (B4), while least lignin deposition in pathogen challenged plants (BC) favored pathogen attack. This finding helps us in understanding the role of bsAgNPs in controlling *B. sorokiniana* infection in wheat plants. However, a more detailed study is required to ascertain this role of bsAgNPs using culture supernatant of *Serratia* sp. BHU-S4.

## Conclusions

Present study encompasses the pivotal role of AgNPs synthesized by an agriculturally important bacterium, *Serratia* sp. BHU-S4 for plant disease control. The bsAgNPs exhibited strong antifungal activity against *Bipolaris sorokiniana* and successfully controlled its infection in wheat plants. The biocontrol potential of bsAgNPs was found to be promising under both *in vitro* and *in vivo* conditions as depicted in the graphical abstract (Figure S1). The foregoing data clearly reveals the robust application of bsAgNPs in agriculture, particularly for plant disease management. Till date many studies have reported antifungal activity of AgNPs under *in vitro* conditions but little is known about its activity under *in vivo* condition. In this regard, our findings certainly authenticate the application of bsAgNPs in controlling plant diseases, thereby pointing to the exciting possibilities of nanofungicide. However, further research is required to verify the effect of bsAgNPs on different phytopathogens causing serious crop losses under field conditions. Moreover, future studies should also be employed for developing efficient delivery systems for large scale application of AgNPs.

## Supporting Information

Figure S1 **Graphical abstract of the study.**
(TIF)
