# Supplementary figures and images for: Biofabricated Silver Nanoparticles Act as a Strong Fungicide against Bipolaris sorokiniana Causing Spot Blotch Disease in Wheat
Source: PLoS One. 2014 May 19;9(5):e97881. doi: 10.1371/journal.pone.0097881 (PMC4026416; doi:10.1371/journal.pone.0097881)

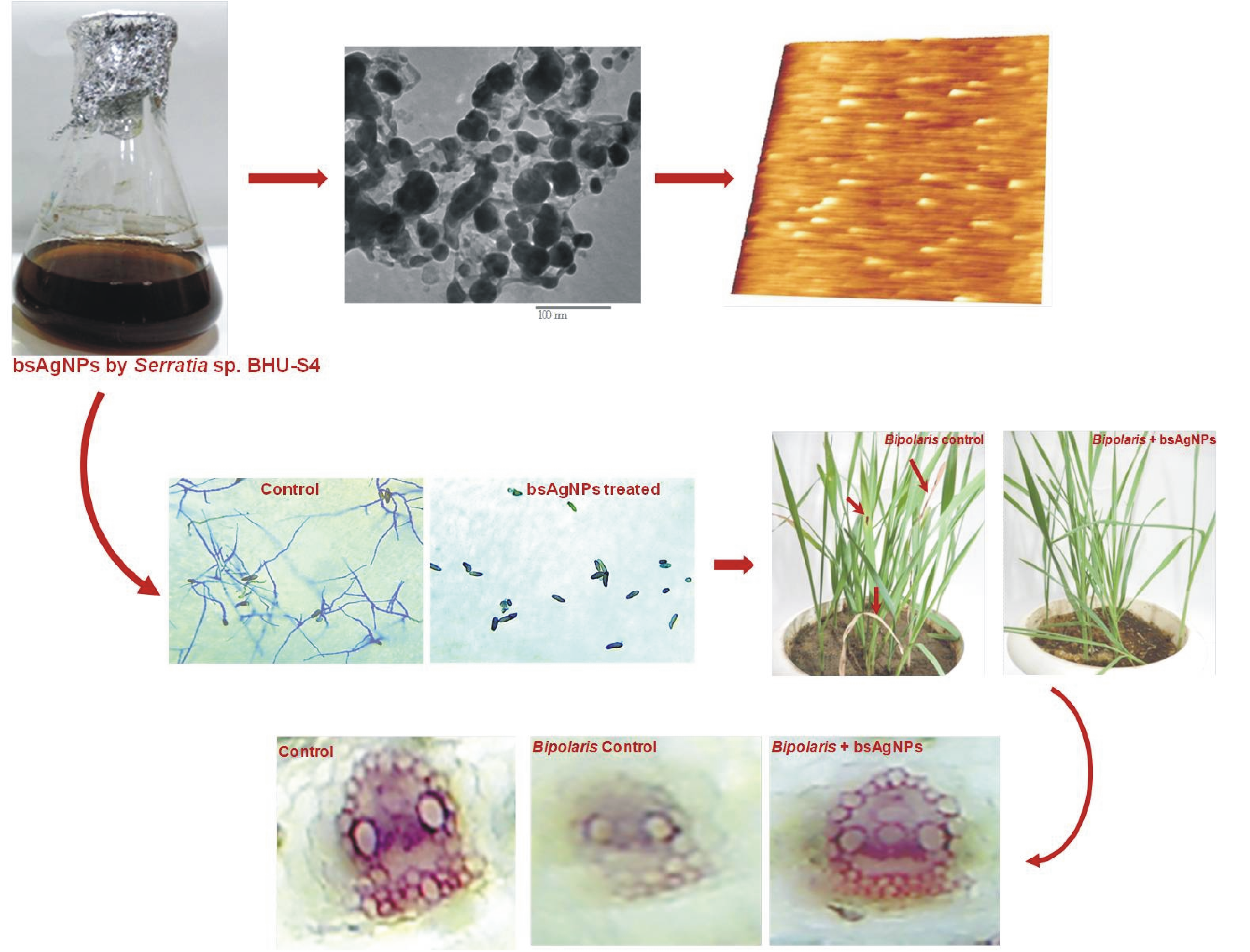

Supplement: Figure S1 — Graphical abstract of the study. (TIF) [file pone.0097881.s001.tif]
